# Supplementary material for: Methyl groups as widespread Lewis bases in noncovalent interactions
Source: Nat Commun. 2021 Aug 19;12:5030. doi: 10.1038/s41467-021-25314-y (PMC8376930; doi:10.1038/s41467-021-25314-y)
Supplement: Supplementary file 1 — Supplementary Information [file 41467_2021_25314_MOESM1_ESM.pdf]

Supplementary Information

Methyl groups as widespread Lewis bases in noncovalent interactions

Oliver Loveday[a] and Jorge Echeverría[a,b]*

a) Departament de Química Inorgànica i Orgànica b) Institut de Química Teòrica i Computacional IQTC-UB, Universitat de Barcelona, Martí i Franquès 1-11, 08028 Barcelona (Spain).

e-mail: jorge.echeverria@qi.ub.es

Supplementary Table 1. Selected examples of noncovalent interactions with a methyl group as the electron density donor. ρ_{BCP} is the value of the electron density (a.u.) at the donor-acceptor BCP, and d_{vdW} is the vdW-corrected interatomic distance in Å.

Bond type	Donor (Y)	Acceptor ($E-CH_3$)	CSD Refcode	E-C···Y angle	d_{vdW} (C···Y)	ρ_{BCP}
Alkaline	R-**Li**-R	$Si-CH_3$	gudgao	174.2	-1.367	0.0077
Alkaline	**Na^+**	Al^--CH_3	cuvmah	179.5	-1.065	0.0088
Alkaline earth	R-**Mg**-R	$Si-CH_3$	jejgio	172.3	-1.745	0.0164
Triel	**Ga**R_3	$Ga-CH_3$	ofuruc	167.2	-0.941	0.0080
Tetrel	**Pb**R_3	$Si-CH_3$	kapteb	171.0	-0.526	0.0047
Pnictogen	**Sb**R_3	$C_{sp^3}-CH_3$	omecuf	166.1	-0.641	0.0069
Chalcogen	R-**Se**-R	$C_{sp^3}-CH_3$	zunxis	170.1	+0.017	0.0047
Halogen	C_{sp^2}-**Br**	$C_{sp^2}-CH_3$	xuvsen	175.6	-0.252	0.0064

Supplementary Table 2. NBO second order perturbation energies (kcal mol^{-1}) calculated at the M06-2X/def2-TZVPD level of theory.

**System**	**Charge transfer**	**Energy (kcal mol^{-1})**
CUVMAH	$BD_{C-Al} \rightarrow LP^*_{Na}$ (s)	4.01
	$BD_{C-Al} \rightarrow LP^*_{Na}$ (s)	0.46
	$BD_{C-H} \rightarrow LP^*_{Na}$ (s)	0.22
	$BD_{C-Al} \rightarrow LP^*_{Na}$ (p)	0.24
**1**	$CR_C \rightarrow LP^*_{Li}$	1.76
	$BD_{C-Al} \rightarrow RY^*_{Li}$	0.76
	$BD_{C-H} \rightarrow LP^*_{Li}$	4.85
	$BD_{C-H} \rightarrow LP^*_{Li}$	4.81
	$BD_{C-H} \rightarrow LP^*_{Li}$	4.68
**2**	$BD_{C-Al} \rightarrow LP^*_{Ga}$	1.31
	$BD_{C-H} \rightarrow LP^*_{Ga}$	5.88
	$BD_{C-H} \rightarrow LP^*_{Ga}$	5.96
	$BD_{C-H} \rightarrow LP^*_{Ga}$	5.98
**3b**	$BD_{C-Al} \rightarrow BD^*_{Ge-C}$	0.30
	$BD_{C-H} \rightarrow BD^*_{Ge-C}$	0.42
	$BD_{C-H} \rightarrow BD^*_{Ge-C}$	0.41
	$BD_{C-H} \rightarrow BD^*_{Ge-C}$	0.44

**4b**	$BD_{C\text{-}Al} \rightarrow BD^{*}_{As\text{-}C}$	0.37
	$BD_{C\text{-}H} \rightarrow BD^{*}_{As\text{-}C}$	0.39
	$BD_{C\text{-}H} \rightarrow BD^{*}_{As\text{-}C}$	0.34
	$BD_{C\text{-}H} \rightarrow BD^{*}_{As\text{-}C}$	0.18
**5b**	$BD_{C\text{-}Al} \rightarrow BD^{*}_{Se\text{-}C}$	0.36
	$BD_{C\text{-}H} \rightarrow BD^{*}_{Se\text{-}C}$	0.11
	$BD_{C\text{-}H} \rightarrow BD^{*}_{Se\text{-}C}$	0.28
	$BD_{C\text{-}H} \rightarrow BD^{*}_{Se\text{-}C}$	0.39
**6b**	$BD_{C\text{-}Al} \rightarrow BD^{*}_{Br\text{-}C}$	0.37
	$BD_{C\text{-}H} \rightarrow BD^{*}_{Br\text{-}C}$	0.25
	$BD_{C\text{-}H} \rightarrow BD^{*}_{Br\text{-}C}$	0.23
	$BD_{C\text{-}H} \rightarrow BD^{*}_{Br\text{-}C}$	0.22

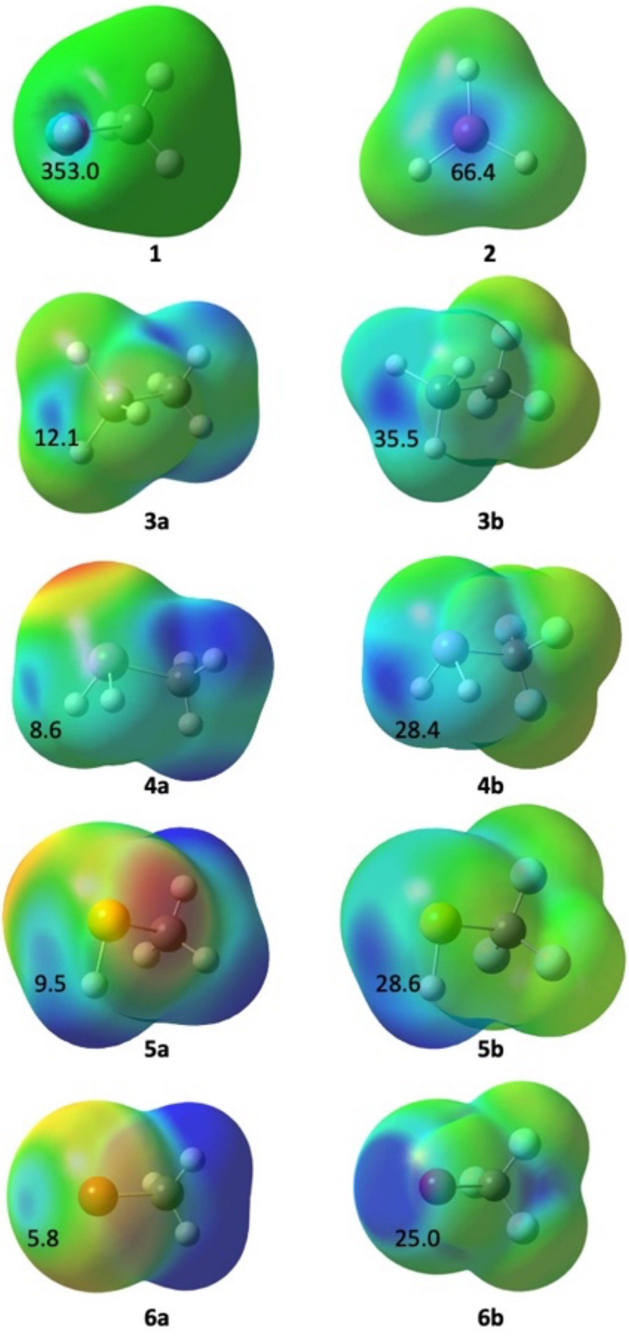

Supplementary Figure 1. MEP maps of the Lewis acids in **1**, **2**, **3a** - **6a**, **3b** - **6b**. EP values are given in kcal/mol. Red and blue represent negative and positive MEP regions, respectively.

Supplementary Table 3. Cartesian coordinates of optimized system **1**.

C	7.253196	8.547783	1.272359
Al	6.406419	10.153849	0.486659
C	7.214019	10.901532	-1.116622
C	4.826142	10.854746	1.377613
H	7.845030	8.028066	0.512626

H	6.478087	7.892714	1.683455
H	7.908537	8.888374	2.083837
H	4.572233	11.866087	1.058062
H	4.942172	10.859289	2.463964
H	3.960596	10.219881	1.163222
H	8.283431	11.078210	-0.973505
H	6.756659	11.840873	-1.428584
H	7.129868	10.198932	-1.951065
Li	8.248385	6.733032	2.213167
C	9.156763	5.199468	3.069290
H	10.055458	4.883816	2.525336
H	9.482251	5.417609	4.093776
H	8.514928	4.312507	3.136580

Supplementary Table 4. Cartesian coordinates of optimized system **2**.

C	7.346016	8.564749	1.363369
Al	6.510363	10.155124	0.551584
C	6.776031	10.469508	-1.352562
C	5.446788	11.330170	1.683957
Ga	8.566402	6.357473	2.415322
H	6.999034	8.435841	2.390111
H	8 .429770	8.718784	1.339355
H	7.090604	7.696176	0.749436
H	5.896272	10.134634	-1.911645
H	7.635215	9.931275	-1.755694
H	6.899444	11.531011	-1.577533
H	4.627291	10.779107	2.153777
H	5.016740	12.174532	1.144136
H	6.053036	11.730050	2.501902
H	9.377017	6.035086	1.090885
H	9 .268072	7.170586	3.582154
H	7.197603	5.607705	2.696635

Supplementary Table 5. Cartesian coordinates of optimized system **3a**.

C	7.144197	8.780346	1.240679
Al	6.294440	10.326626	0.397242
C	4.899754	11.311415	1.348812
C	6.840362	10.884808	-1.394655
Ge	8.783069	5.913452	2.693993
C	9.669034	4.352592	3.496246
H	8.224876	8.930674	1.325724
H	7.009835	7.882880	0.628959
H	6.757439	8.565481	2.238243
H	4.498647	12.152940	0.782377
H	5.279213	11.698839	2.298926
H	4.064962	10.651649	1.603034
H	7.625518	10.260037	-1.822569
H	7.202537	11.917047	-1.385571
H	5.989154	10.868833	-2.081660
H	10.740447	4.526872	3.584200
H	9.268535	4.156462	4.489805
H	9.512666	3.468176	2.880298
H	9.360189	6.181557	1.284128

H	7.262879	5.654742	2.573224
H	9.012208	7.163170	3.576148

Supplementary Table 6. Cartesian coordinates of optimized system **3b**.

C	7.155981	8.760874	1.221606
Al	6.269596	10.277165	0.349302
C	6.761585	10.759217	-1.477485
C	4.906492	11.281482	1.321057
Ge	8.752306	6.076746	2.693910
C	9.688711	4.512397	3.566235
H	8.243250	8.875026	1.177767
H	6.916343	7.832703	0.692648
H	6.862269	8.642229	2.266172
H	5.319858	11.707117	2.240074
H	4.088926	10.624677	1.632257
H	4.474989	12.098010	0.740743
H	7.490802	10.081630	-1.923524
H	7.184820	11.767775	-1.507094
H	5.881794	10.780906	-2.126623
F	11.028274	4.638768	3.535412
F	9.347063	4.370649	4.860161
F	9.400152	3.344221	2.963480
H	9.239485	6.104784	1.236747
H	7.247130	5.789649	2.809569
H	9.180136	7.322574	3.485170

Supplementary Table 7. Cartesian coordinates of optimized system **4a**.

C	7.311262	8.822014	1.229947
Al	6.319213	10.258946	0.349134
C	4.727553	10.987545	1.219951
C	6.897308	10.975133	-1.375179
As	9.192864	6.231277	2.849968
C	9.587148	4.403035	3.500185
H	8.347327	8.752945	0.892560
H	6.839453	7.856526	1.016131
H	7.312788	8.932225	2.317057
H	5.019305	11.643972	2.046283
H	4.109904	10.200195	1.658963
H	4.101291	11.575644	0.547263
H	7.741122	10.435701	-1.807389
H	7.195080	12.023148	-1.270442
H	6.080371	10.963621	-2.101797
H	10.538765	4.083824	3.079622
H	9.684029	4.434153	4.583678
H	8.813145	3.692049	3.225898
H	8.805429	5.817164	1.434775
H	7.739993	6.256541	3.311017

Supplementary Table 8. Cartesian coordinates of optimized system **4b**.

C	7.284269	8.849105	1.312168
Al	6.292420	10.258016	0.376346
C	6.848620	10.810372	-1.411895

C	4.759604	11.086801	1.257529
As	9.100195	6.380858	2.913731
C	9.591552	4.497849	3.430407
H	8.355458	8.905490	1.102677
H	6.941514	7.868153	0.964708
H	7.137333	8.883900	2.393666
H	5.072757	11.605768	2.168368
H	4.034010	10.331717	1.573042
H	4.238159	11.808851	0.627861
H	7.542815	10.111455	-1.880901
H	7.351806	11.781616	-1.363092
H	5.993568	10.942224	-2.079104
F	10.858020	4.228321	3.080957
F	9.504672	4.335257	4.758829
F	8.825255	3.552752	2.872487
H	9.006167	5.997594	1.445468
H	7.625156	6.109561	3.164374

Supplementary Table 9. Cartesian coordinates of optimized system **5a**.

C	-1.14658	-0.48734	-0.07299
Al	-3.04487	-0.02225	-0.01182
C	-4.39209	-1.43789	0.04598
C	-3.59156	1.85383	-0.00999
H	-0.96650	-1.55792	-0.18499
H	-0.64800	-0.16167	0.84616
H	-0.64214	0.03054	-0.89422
H	-4.16859	2.08553	-0.91050
H	-2.75104	2.54794	0.03089
H	-4.24973	2.07111	0.83586
H	-4.32883	-2.06673	-0.84715
H	-5.41370	-1.06171	0.11509
H	-4.22196	-2.10335	0.89728
Se	2.36547	0.14899	0.03248
C	4.26675	-0.30433	-0.04476
H	4.51755	-0.72177	-1.01464
H	4.80526	0.62921	0.09674
H	4.52487	-0.99416	0.75233
H	1.88089	-1.21905	-0.18296

Supplementary Table 10. Cartesian coordinates of optimized system **5b**.

C	2.11604	0.12989	-0.04822
Al	4.07305	0.04683	0.02355
C	5.10878	1.70191	-0.01189
C	4.96064	-1.68922	0.12672
H	1.73749	1.14327	-0.19345
H	1.68792	-0.26802	0.87763
H	1.73828	-0.49601	-0.86240
H	5.53512	-1.87753	-0.78556
H	4.26819	-2.52204	0.25647
H	5.68020	-1.71673	0.94937
H	4.86629	2.29483	-0.89838
H	6.18708	1.53784	-0.00385
H	4.86195	2.32965	0.84959
Se	-1.27960	-0.25735	-0.15211

C	-3.19269	0.11506	0.07550
F	-3.58111	1.21603	-0.56647
F	-3.88642	-0.91132	-0.41568
F	-3.53732	0.26805	1.35342
H	-0.91890	1.01521	0.48232

Supplementary Table 11. Cartesian coordinates of optimized system **6a**.

C	7.246706	8.688134	1.205507
Al	6.349115	10.238474	0.424045
C	6.941127	10.944613	-1.300334
C	4.858696	11.084489	1.365033
H	8.045926	8.289119	0.578772
H	6.533428	7.880042	1.394205
H	7.680478	8.942298	2.177674
H	5.062284	12.144299	1.543669
H	4.634754	10.620478	2.326537
H	3.948999	11.049089	0.758164
H	7.993759	11.239401	-1.256594
H	6.366856	11.809021	-1.636453
H	6.874312	10.176600	-2.076528
Br	8.762294	5.944371	2.688068
C	9.597906	4.407122	3.524015
H	10.663581	4.461650	3.333380
H	9.391189	4.451938	4.587130
H	9.170237	3.514007	3.083078

Supplementary Table 12. Cartesian coordinates of optimized system **6b**.

C	7.234475	8.685600	1.234205
Al	6.346527	10.235738	0.430162
C	6.998634	10.949485	-1.266859
C	4.817901	11.051108	1.332024
H	8.062608	8.309606	0.630831
H	6.519291	7.870369	1.381008
H	7.622271	8.941916	2.225102
H	5.001115	12.111798	1.526226
H	4.571011	10.575248	2.282001
H	3.927884	11.008711	0.697222
H	8.046257	11.252886	-1.182373
H	6.430304	11.810237	-1.621679
H	6.967128	10.183567	-2.047253
Br	8.715357	6.025045	2.655012
C	9.563110	4.493379	3.465513
F	10.746082	4.275947	2.916040
F	9.732760	4.691689	4.761858
F	8.818931	3.412817	3.300327
